# Polymorphisms and NIHL: a systematic review and meta-analyses

**DOI:** 10.3389/fncel.2023.1175427

**Published:** 2023-06-15

**Authors:** Lu Wang, HanYu Wang, Feng Xiang, YuLu Xiang, Feng Xiong, QinXiu Zhang

**Affiliations:** ^1^Clinical Medical College, Chengdu University of Traditional Chinese Medicine, Chengdu, Sichuan, China; ^2^The Affiliated Hospital of Inner Mongolia Medical University, Hohhot, China; ^3^Hospital of Chengdu University of Traditional Chinese Medicine, Chengdu, China

**Keywords:** noise induced hearing loss, polymorphism, meta-analysis, GRHL2, CAT, EYA4, HSP70

## Abstract

**Background:**

Noise-induced hearing loss (NIHL) has always been a global public health problem, which is related to noise exposure and genetic factors. Many researchers have tried to identify the polymorphisms that cause different individuals' susceptibility to NIHL. We conducted a meta-analysis of the most frequently studied polymorphisms to identify those genes that may be associated with NIHL and may provide value in risk prevention.

**Methods:**

PubMed, China National Knowledge Infrastructure (CNKI) database, Embase, Wang Fang, Web of Science and Cochrane library were searched, and qualified studies on the correlation between polymorphism and NIHL susceptibility were screened, and then polymorphisms cited in at least three studies were selected for meta-analysis. Fixed- or random-effects models were used to calculate odds ratios and 95% confidence intervals. Statistical I^2^ tests and sensitivity analyses were used to detect interstudy heterogeneity and test the statistical stability of overall estimates, respectively. Egger's tests were applied to detect publication bias among included studies. All of the above analyses were performed using stata 17.0.

**Results:**

64 genes were initially selected and introduced in 74 papers. Among them, 10 genes (and 25 polymorphisms) have been reported in more than 3 papers. Twenty five polymorphisms participated in the meta-analysis. Of the 25 polymorphisms, only 5 were significantly associated with the risk of AR: rs611419 (GRHL2) polymorphism and rs3735715 polymorphism (GRHL2), rs208679 polymorphism (CAT), rs3813346 polymorphism (EYA4) were significantly associated with the susceptibility of NIHL, rs2227956 polymorphism (HSP70) was significantly associated with the susceptibility of white population NIHL, and the remaining 20 gene polymorphisms were not significantly associated with NIHL.

**Conclusion:**

We found polymorphisms that are valuable for the prevention of NIHL and polymorphisms that are not related to NIHL. This is the first step to establish an effective risk prediction system for the population, especially for high-risk groups, which may help us better identify and prevent the occurrence of NIHL. In addition, our research results contribute to the in-depth exploration of NIHL.

**Systematic review registration:**

https://inplasy.com/inplasy-2023-6-0003/, identifier INPLASY202360003.

## Introduction

Hearing loss is the most common sensory impairment in the world. WHO estimates that by 2050, nearly 2.5 billion people will have different degrees of hearing loss, and at least 700 million people need hearing rehabilitation (Chadha et al., 2021). Noise is the **second** largest cause of adult hearing loss. As a progressive sensorineural hearing loss caused by noise exposure, noise-induced hearing loss (NIHL) has always been a global public health problem (Sliwińska-Kowalska and Zaborowski, 2017; Chadha et al., 2021). Noise-induced deafness generally develops into an irreversible lesion through several stages, including auditory adaptation, auditory fatigue, temporary threshold shift and permanent threshold shift (Zhu et al., 2016). At present, there are few effective clinical treatments for NIHL, which is an incurable but completely preventable disease (Masterson et al., 2016). After a lot of research, we have clearly known that the degree of hearing loss in NIHL will increase with the increase of noise intensity and exposure duration. Secondly, individual susceptibility to NIHL varies greatly. We found that different individuals exposed to very similar noise occupational environment showed different degrees of NIHL (Hong et al., 2013). The mainstream idea is that individuals' various susceptibility to the same noise exposure may be due to different genetic backgrounds. We all know that genetic factors will affect individual susceptibility to noise in animal models (Erway et al., 1996). But whether this conclusion can be directly applied to humans is controversial. In order to explain the differences in susceptibility of different individuals and provide new methods to predict the risk of NIHL, exploring the individual susceptibility of NIHL from the level of gene polymorphism has become the point of penetration for many researchers to explore hearing loss and has achieved certain results. This inconsistency may result from the differences in sample characteristics (such as regional differences, racial differences, gender composition differences, age composition differences), small sample size and single sample source of these studies. Therefore, some researchers have explored the gene polymorphisms that are significantly related to NIHL through comprehensive evaluation, which is conducive to formulating more targeted NIHL precaution and control strategies. However, just to systematically evaluate the association between single nucleotide polymorphisms and NIHL in single genes, which is far away from to establish a practical prediction system for individual susceptibility to NIHL. Chen et al. classified a large group of genes that have been found to be associated with NIHL according to the pathogenesis of NIHL (Chen et al., 2022), but such retrospective review did not see data analysis. Therefore, the purpose of this study was to review the research on the relationship between SNP and NIHL susceptibility in detail and systematically through a large number of retrieval of relevant documents, and carry out meta-analysis of SNP to solve the results of these contradictions, and look forward to finding out the genes or SNPs associated with NIHL susceptibility, So as to establish a practical and reliable prediction system. With the rapid growth of biomedical literature, there is increasing need to make meaningful inferences from a comprehensive and complex body of evidence, a thorough review and meta-analysis of the literature helps to explore more evidence of correlation between NIHL incidence and gene polymorphism.

## Methods

### Search strategy

We comprehensively searched PubMed, CNKI, Embase, Wang Fang, Web of Science and Cochrane library. The key words are (“noise induced hearing loss” or “NIHL”) and (“gene polymorphism” or SNP). The detailed retrieval strategy is shown in Supplementary Table S1. We did not set the retrieval time and the last retrieval time was updated on February 18, 2023.

### Inclusion and exclusion criteria

First, we will de-duplicate all the documents we have retrieved, and then remove the documents irrelevant to NIHL or gene polymorphism by reading the title and abstract of the article. Finally, we will read the full text of the preliminary screening documents and include the final documents to be analyzed in strict accordance with the inclusion criteria. The inclusion criteria are as follows: (1) the literature on the correlation between gene polymorphism and NIHL susceptibility using case-control study or cross-sectional study, (2) both NIHL case group and control group conform to the HWE equilibrium law, (3) the original data directly or indirectly provide the allele or genotype frequency of each SNP in the case and control group. (4) Two independent sample sets in one study are considered as two different studies. Any one of the following situations will not be included: (1) randomized controlled experimental research, animal experimental research or cell research; (2) Case reports, meta-analysis studies, reviews, books, conferences; (3) Incomplete information or invalid original data; (4) There are <3 studies on a gene or a SNP.

### Data extraction and quality assessment

Each included study was independently reviewed by **two** researchers. Each study collected the following data items: **first** author, year of publication, country (region), characteristics of participants (race, work unit, sample size, age, sex), allele and genotype frequency of SNP in each gene in case and control, gene detection method and other information (Supplementary material S2, S3). In the process of data collection, whenever there is any problem, we will consult other available researchers or refer to relevant literature and books for clarification. The Newcastle-Ottawa Scale (NOS) of the case-control study was used to evaluate the quality of the included study (Hwang et al., 2023) (Supplementary material S2). The total score of 0–5 points belongs to low quality research, and 6–9 points belongs to high quality research. The score was completed by **two** researchers independently, and the differences were discussed and resolved by a third person effect size and accompanying confidence intervals.

### Statistical analysis

Meta-analyses should generally be performed using random-effects model to estimate. Statistical heterogeneity should be assessed with I^2^ statistic, with values over 50% indicating substantial heterogeneity, and publication bias should be assessed by funnel plot asym-metry and Egger's regression test. Because of the large number of NIHL susceptibility related gene polymorphisms we analyzed, in order to improve efficiency, all SNPs only compared the gene distribution differences between the case group and the control group under the allele model. All data analysis was completed using stata17.0 software, and the correlation between SNPs of each gene and NIHL risk was evaluated by combining odds ratio (OR) and 95% confidence interval (95% CI). Use I^2^ before merging publication OR values. Test and evaluate the degree of heterogeneity between studies, when I^2^ = 0, there is no heterogeneity. The larger the I squared, the more heterogeneous it is. The size of heterogeneity determines the choice of research model (Higgins and Thompson, 2002). In our study, When I^2^ <50%, fixed effects model is used, and when I^2^≥50%, random effects model is used. In the study, when the OR value is greater than 1, the lower limit of CI is greater than 1, *p* < 0.05, or when the OR is <1, and the upper boundary of CI is <1, *p* < 0.05, it is considered statistically significant (Zlowodzki et al., 2007). We used Egger's test and funnel plot to estimate the publication bias of objective quantitative estimation and conducted sensitivity analysis to evaluate the stability of the summary results.

## Results

### Search methods and data screening

**Figure 1:**
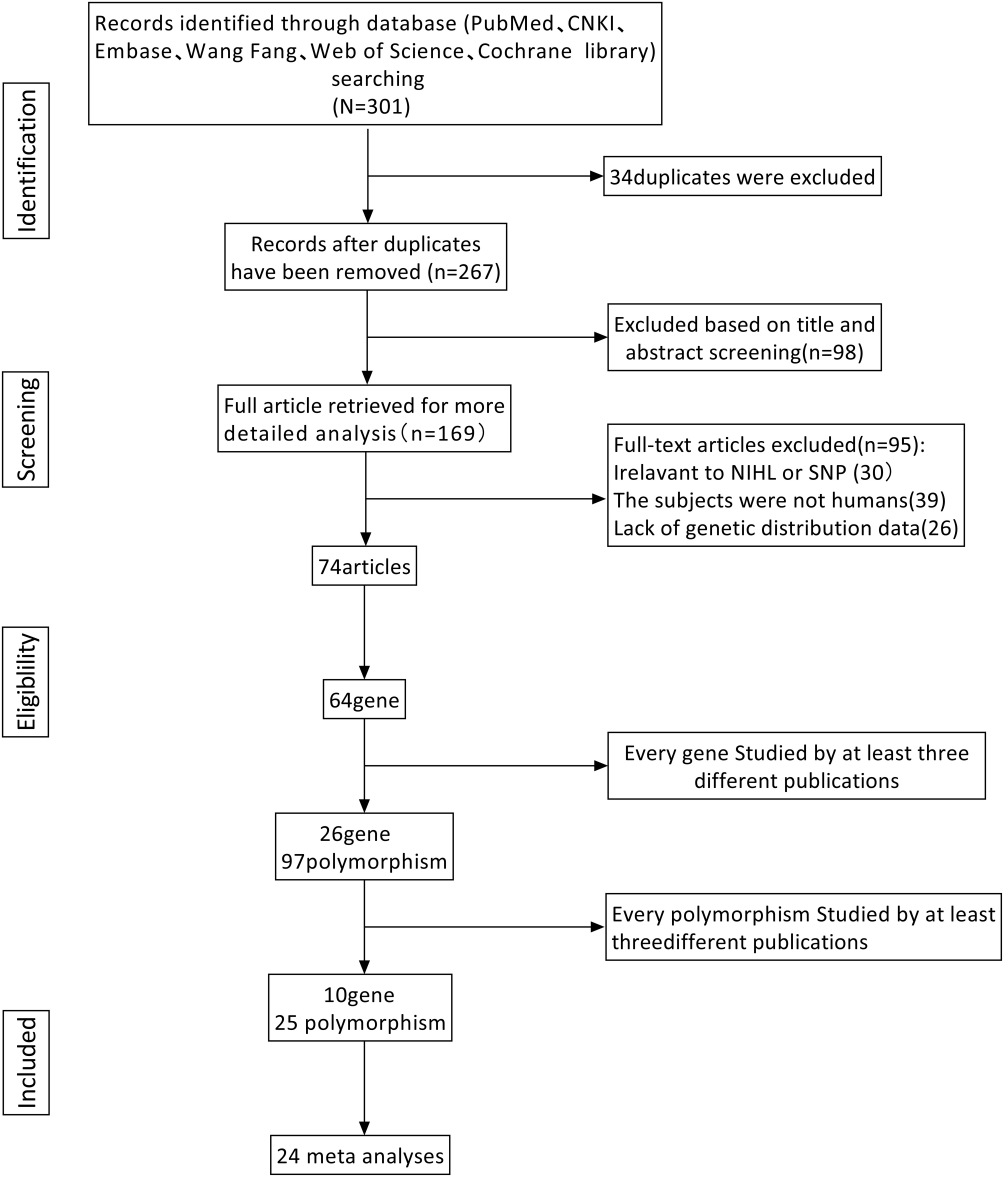
Screening of polymorphisms for meta-analysis.

After screening (Figure 1), a total of 25 polymorphisms reported by at least 3 literatures were selected for meta-analysis. Seventy four papers (Supplementary material S4) reported 64 different genes, 26 of which were studied in at least 3 publications and only 25 polymorphisms were studied in at least 3 publications, so we conducted a systematic review of each of the 25 screening polymorphisms (Table 1). The number of publications including each polymorphism, the degree of heterogeneity, the number of patients and controls, OR **(**95% CI), and the application of random effects or fixed effects models are shown in Table 1.

**Table 1:** The primary results of the meta-analyses in exploring the relationship between gene polymorphisms and NIHL.

**Gene**	**Polymorphisms**	**Minor allele**	**Total studies**	**Ethnicity**	**Number of patient**		**Fixed or random effects model **	**OR**	**CI**	***p*-value**	**Funnel plot asymmetry linear regression test**	
					**NIHL**	**Control**					***t***	***p*** **-value**
CAT	rs208679	G	3	Asian subgroup	1,017	736	Fixed	0.811	(0.673; 0.977)	0.027	−0.72	0.603
CAT	rs769217	T	4	Asian subgroup	1,702	1,462	Fixed	1.018	(0.918; 1.128)	0.736	0.75	0.533
CAT	rs564250	T	3	Asian subgroup	1,208	1,237	Fixed	1.141	(0.985; 1.321)	0.079	0.73	0.597
CAT	rs769214	A	3	Asian subgroup	1,067	1,417	Fixed	0.884	(0.780; 1.001)	0.052	−0.59	0.663
GST	M1 (m^*^)	null	5	Mixed population	508	601	Random	0.776	(0.509; 1.184)	0.239	0.41	0.707
GST	M1 (a^*^)	null	3	Asian subgroup	377	385	Random	0.706	(0.428; 1.165)	0.173	0.41	0.707
GST	T1 (m^*^)	null	5	Mixed population	508	601	Random	0.982	(0.763; 1.263)	0.886	−0.82	0.470
GST	T1 (a^*^)	null	3	Asian subgroup	377	385	Random	0.976	(0.640; 1.489)	0.910	−0.82	0.470
GSTP1	rs1695	G	3	Asian subgroup	598	1,211	Fixed	1.170	(0.997; 1.375)	0.055	−2.66	0.229
PON2	rs7493	G	3	Asian subgroup	601	622	Random	1.195	(0.353; 4.049)	0.774	0.25	0.844
PON2	rs12026	G	3	Asian subgroup	601	622	Random	1.038	(0.325; 3.318)	0.95	0.53	0.688
PON2	rs7785846	T	4	Mixed population	723	788	Random	1.042	(0.438; 2.480)	0.926	0.88	0.472
					**NIHL**	**Control**					***t***	***p*** **-value**
PON2	rs7786401	T	3	Asian subgroup	601	622	Random	1.132	(0.339; 3.778)	0.841	0.41	0.750
SOD2	rs4880	C	3	Asian subgroup	545	1,038	Random	1.187	(0.846; 1.666)	0.322	0.95	0.517
CDH23	rs1227049	G	7	Asian subgroup	1,105	2,334	Random	1.078	(0.899; 1.292)	0.418	0.21	0.841
CDH23	rs3802711	C	5	Asian subgroup	931	1,254	Random	0.828	(0.616; 1.111)	0.208	−1.90	0.153
CDH23	rs1227051	C	4	Asian subgroup	367	1,275	Fixed	0.973	(0.750; 1.264)	0.84	0.61	0.603
HSP70	rs1043618 (m^*^)	C	6	Mixed population	930	1,243	Fixed	1.095	(0.958; 1.252)	0.182	0.61	0.573
HSP70	rs1043618 (c^*^)	C	2	Caucasian subgroup	217	227	Fixed	1.278	(0.964; 1.694)	0.088	0.61	0.573
HSP70	rs1043618 (a^*^)	C	4	Asian subgroup	713	1,016	Fixed	1.047	(0.900; 1.219)	0.551	0.61	0.573
HSP70	rs2227956 (m^*^)	C	5	Mixed population	903	921	Random	0.839	(0.631; 1.114)	0.224	−1.56	0.216
HSP70	rs2227956 (c^*^)	C	2	Caucasian subgroup	217	227	Random	0.535	(0.368; 0.779)	0.001	−1.56	0.216
HSP70	rs2227956 (a^*^)	C	3	Asian subgroup	687	694	Random	1.026	(0.840; 1.254)	0.799	−1.56	0.216
HSP70	rs2763979	T	3	Asian subgroup	620	915	Random	0.982	(0.712; 1.356)	0.914	−0.88	0.540
CASP3	rs1049216	T	3	Asian subgroup	851	851	Random	0.857	(0.542; 1.354)	0.508	−89.36	0.007
					**NIHL**	**Control**					***t***	***p*** **-value**
CASP3	rs6948	C	3	Asian subgroup	851	851	Random	0.891	(0.611; 1.299)	0.548	−3.24	0.191
EYA4	rs3813346	T	3	Asian subgroup	961	1,253	Fixed	1.392	(1.230; 1.575)	0.000	0.35	0.783
GRHL2	rs611419	A	3	Asian subgroup	865	881	Fixed	1.157	(1.012; 1.323)	0.033	−1.68	0.342
GRHL2	rs3735715	A	4	Asian subgroup	1,208	1,224	Fixed	0.866	(0.774; 0.968)	0.011	−1.08	0.394
GRHL2	rs3735713	A	3	Asian subgroup	865	881	Fixed	0.999	(0.863; 1.157)	0.991	1.24	0.433
GRHL2	rs3735714	T	3	Asian subgroup	865	881	Fixed	0.961	(0.838; 1.104)	0.576	0.39	0.762

m: Mixed population; a: Asian subgroup; c: Caucasian subgroup.

### Meta-analysis results

#### Antioxidant genes

##### Catalase

**Figure 2:**
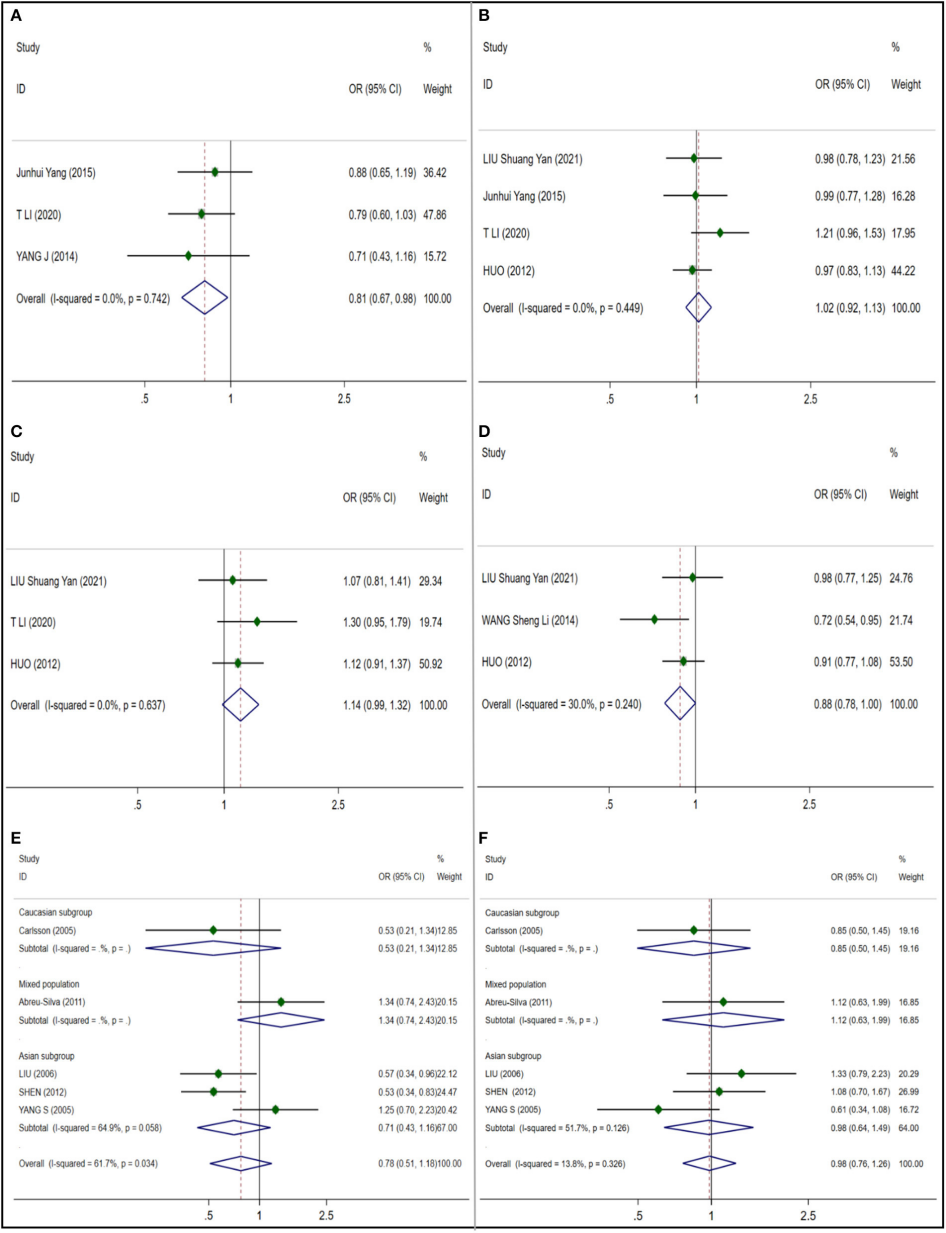
Forest plots for CAT and GST polymorphisms. **(A)** CAT (rs208679): allele G; **(B)** CAT (rs769217): allele T; **(C)** CAT (rs564250): allele T; **(D)** CAT (rs769214): allele A; **(E)** GSTM1: null; **(F)** GSTT1:null.

We studied and analyzed four single nucleotide polymorphisms in CAT gene. And because of the low heterogeneity, the random effect model is adopted. A significant correlation (OR 0.811; 95% CI 0.673;0.977) was found in rs208679 (Figure 2A) which included 1017 NIHL cases and 736 control cases, and no publication bias was observed (*t* = −0.72; *p* = 0.603) (Supplementary Figure S1A). Four articles which included a total of 1702 NIHL cases and 1462 control cases were included in the rs769217 (Figure 2B) analysis, no significant correlation was observed (OR 1.018; 95% CI 0.918;1.128), and no publication bias was observed (*t* = 0.75, *p* = 0.533) (Supplementary Figure S1B). No significant correlation (OR 1.141; 95% CI 0.985;1.321) and publication bias (*t* = 0.73, *p* = 0.597) (Supplementary Figure S1C) were observed in 3 articles included in rs564250 (Figure 2C) which included 1208 NIHL cases and 1237 control cases. Three articles were included in the analysis of rs769214 (Figure 2D) which included 1067 NIHL cases and 1417 control cases, and no significant correlation (OR 0.884; 95% CI 0.780;1.001) was observed, and no publication bias was observed (*t* = −0.59, *p* = 0.663) (Supplementary Figure S1D).

##### Glutathione S-transferase (GST), glutathione S-transferase M1and T1

We included five literature studies to analyze the close relationship between M1 and T1 polymorphisms in GST and susceptibility to NIHL. In the analysis of GSTM1 (Figure 2E), we found that I^2^ = 61.7%, and the heterogeneity was large, so we did a subgroup analysis according to ethnicity, and still found a large heterogeneity in the Asian subgroup, so we adopted the random effect model. And no significant correlation between GSTM1 and NIHL was found in Mixed population (OR 0.776 95% CI 0.509–1.184) (NIHL cases: 508; control cases: 601) and Asian subgroup (OR 0.706; 95% CI 0.428–1.165) (NIHL cases: 377; control cases: 385), and no publication bias was observed (*t* = 0.41; *p* = 0.707) (Supplementary Figure S2A). In the analysis of 5 articles included by GSTT1 (Figure 2F), I^2^ = 13.8%, considering the possible ethnic differences, we still conducted a subgroup analysis according to ethnic groups, I^2^ = 51.7% in the Asian subgroup, so we adopted the random effect model, in the mixed race (OR 0.982; 95% CI 0.763–1.263) (NIHL cases: 508; control cases: 601) and the Asian subgroup (OR 0.976; 95% CI 0.640–1.489) (NIHL cases: 377; control cases: 385). No significant correlation between GSTM1 and NIHL was found in 95%CI 0.640–1.489), and no publication bias was observed (*t* = −0.82; *p* = 0.470) (Supplementary Figure S2B).

##### Glutathione S-transferase P1

**Figure 3:**
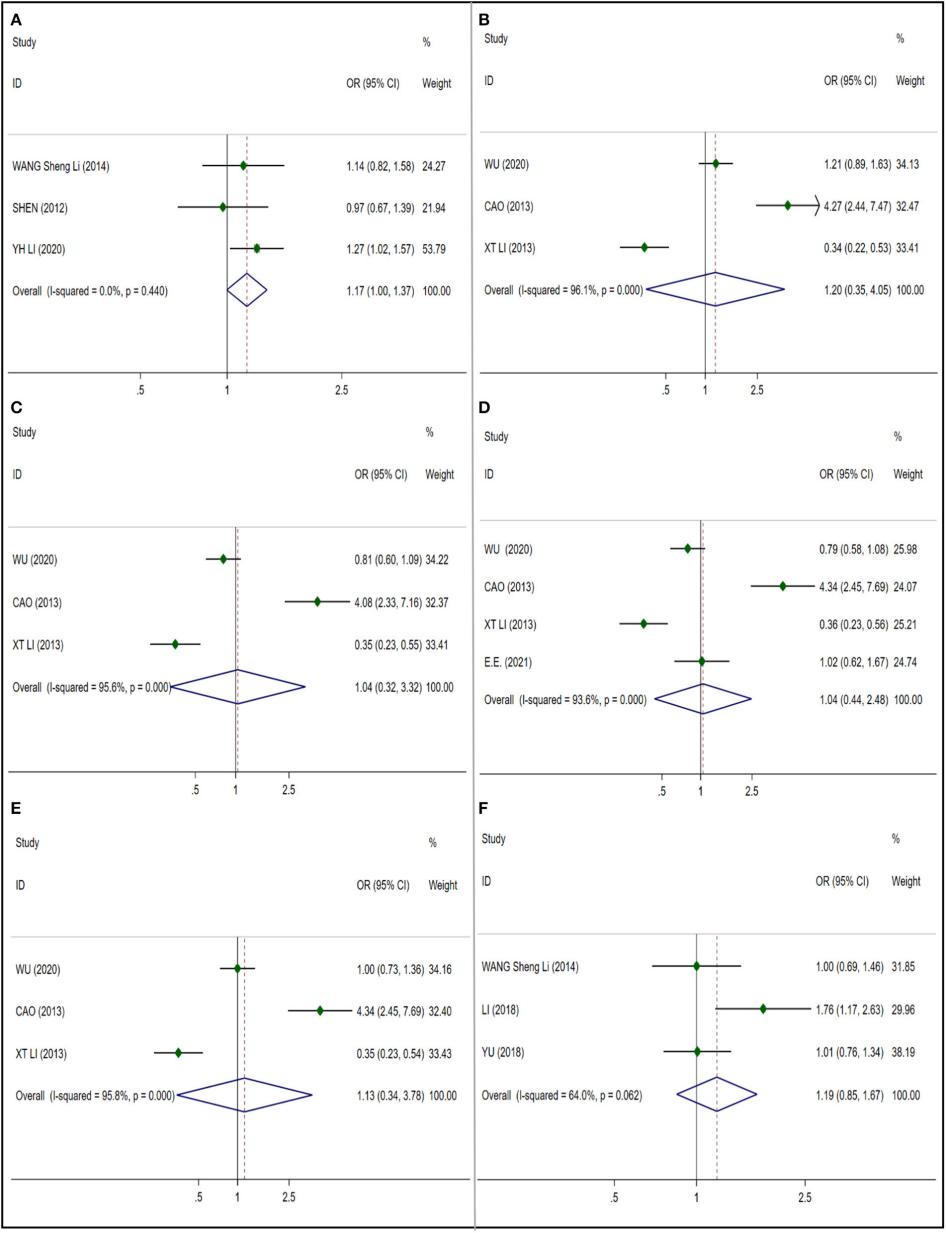
Forest plots for GSTP1,PON2and SOD2 polymorphisms. **(A)** GSTP1 (rs1695): allele G; **(B)** PON2 (rs7493): allele G; **(C)** PON2 (rs12026): allele G; **(D)** PON2 (rs7785846): allele T; **(E)** PON2 (rs7786401): allele T; **(F)** SOD2 (rs4880): allele C.

We selected only one gene polymorphism rs1695 (Figure 3A) (NIHL cases: 598; control cases: 1,211) of GSTP1 for meta-analysis, and used the fixed effect model. There is no significant correlation was found (OR 1.170; 95% CI 0.997–1.375), and no publication bias was observed (*t* = −2.66; *p* = 0.229) (Supplementary Figure S2C).

##### Paraoxonase-2

We analyzed four gene polymorphisms of PON2: rs7493, rs12026, rs7785846 and rs7786401. We included three literature studies in rs7493 (Figure 3B) which included 601 NIHL cases and 622 control cases, using random effect model, and found no significant correlation (OR 1.195; 95% CI 0.353–4.049), and no publication bias was observed (*t* = 0.25; *p* = 0.844) (Supplementary Figure S3A). We included three literature studies in rs12026 (Figure 3C)which included 601 NIHL cases and 622 control cases, using random effect model, and found no significant correlation (OR 1.038; 95% CI 0.325–3.318), and no publication bias was observed (*t* = 0.53; *p* = 0.688) (Supplementary Figure S3B). Among them, rs7785846 (Figure 3D) (NIHL cases: 723; control cases: 788) we included four literature studies for analysis, using random effect model, no significant correlation was found (OR 1.042; 95% CI 0.438–2.480), and no publication bias was observed (*t* = 0.88; *p* = 0.472) (Supplementary Figure S3C). Three literature studies were included in rs7786401 (Figure 3E) which included 601 NIHL cases and 622 control cases, using random effect model, no significant correlation was found (OR 1.132; 95% CI 0.339–3.778), and no publication bias was observed (*t* = 0.41; *p* = 0.750) (Supplementary Figure S3D).

##### Superoxide dismutase 2

According to the number of documents included. We selected only one gene polymorphism rs4880 (Figure 3F) of SOD2 which included 545 NIHL cases and 1038 control cases to analyze, using random effect model, no significant correlation was found (OR 1.187; 95% CI 0.846–1.666) and no publication bias was observed (*t* = 0.95; *p* = 0.517) (Supplementary Figure S4A).

#### Cilia structure related genes

##### Cadherin related 23

**Figure 4:**
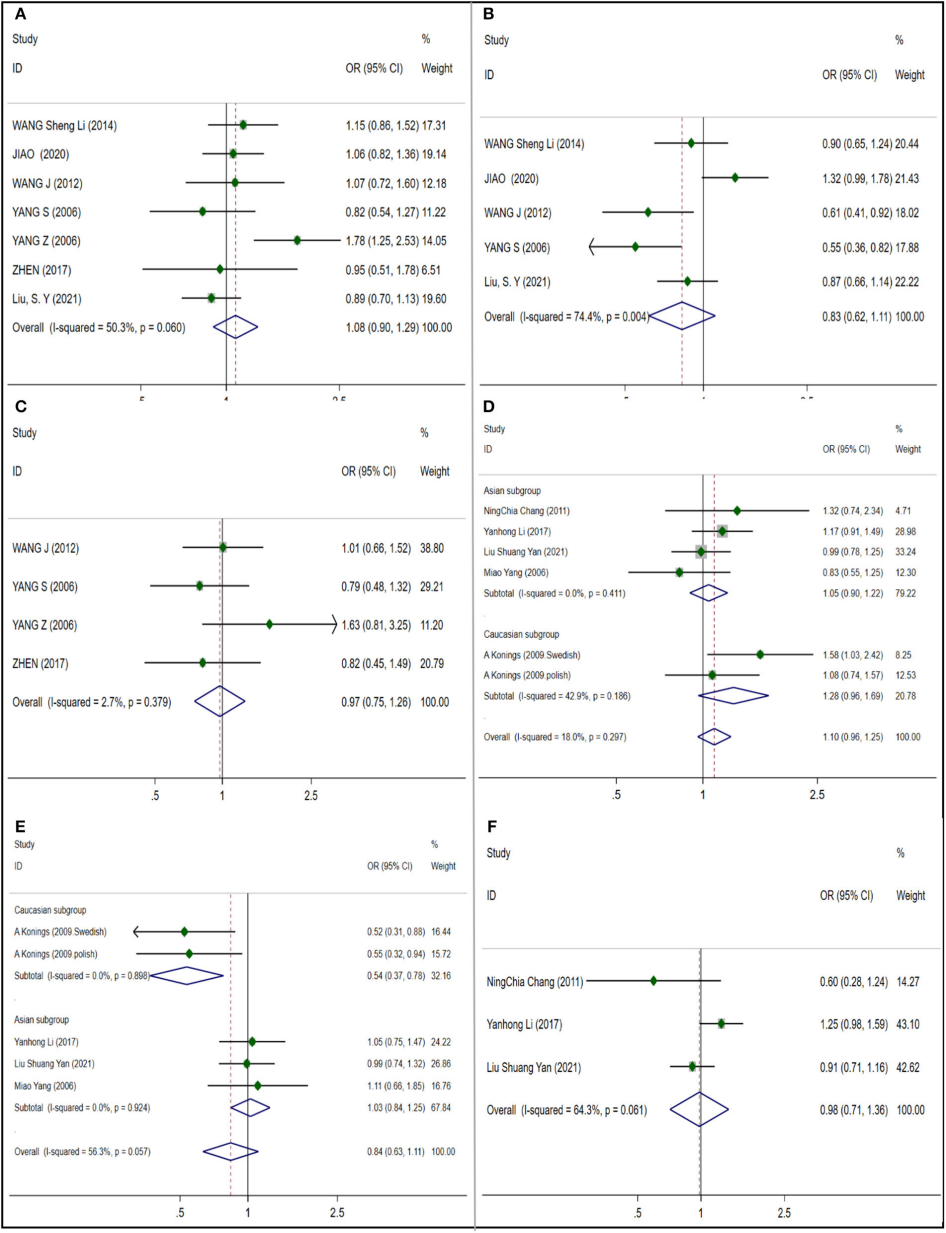
Forest plots for CDH23 and CDH23 polymorphisms. **(A)** CDH23 (rs1227049): allele G; **(B)** CDH23 (rs3802711): allele C; **(C)** CDH23 (rs1227051): allele C; **(D)** HSP70 (rs1043618): allele C; **(E)** HSP70 (rs2227956): allele T; **(F)** HSP70 (rs2763979): allele T.

We analyzed the significant relationship between three gene polymorphisms rs1227049, rs3802711, rs1227051 and NIHL in CDH23. We included 7 articles in rs1227049 (Figure 4A) which included 1,105 NIHL cases and 2,334 control cases and got I^2^ = 50.3%, so we adopted the random effect model. And no significant correlation was observed in the analysis of these literatures (OR 1.078; 95% CI 0.899–1.292) and no publication bias was observed (*t* = 0.21; *p* = 0.841) (Supplementary Figure S5A). The I^2^ = 91.1% of the five articles we included in rs3802711 (Figure 4B) which included 931 NIHL cases and 1254 control cases, the heterogeneity is large, and the random effect model is used. No significant correlation was observed in the analysis (OR 0.828; 95% CI 0.616–1.111) and no publication bias was observed (*t* = −1.90; *p* = 0.153) (Supplementary Figure S5B). We included 4 articles in rs1227051 (Figure 4C) (NIHL cases: 367; control cases: 1,275), and the result was I^2^ = 2.7%, using the fixed effect model. And no significant correlation was observed in the analysis of these four literatures (OR 0.973; 95% CI 0.750–1.264) and no publication bias was observed (*t* = 0.61; *p* = 0.603) (Supplementary Figure S5C).

##### Heat Shock Protein Genes 70

In the analysis of HSP70, only three gene polymorphisms of rs1043618, rs2227956 and rs2763979 were considered in our work. We also conducted a subgroup analysis based on the six studies included in the rs1043618 (Figure 4D), all of which adopted a fixed effect model. No significant differences were found in mixed subgroups (OR 1.095; 95% CI 0.958–1.252) (NIHL cases: 930; control cases: 1,275), Caucasian subgroup (OR 1.278; 95% CI 0.964–1.694) (NIHL cases: 217; control cases: 227) and Asian subgroup (OR 1.047% CI 0.900–1.219) (NIHL cases: 713; control cases: 1,016), and no publication bias was observed (*t* = 0.61; *p* = 0.573) (Supplementary Figure S6A). We included 5 studies in rs2227956 (Figure 4E) and got I^2^ = 56.3%. The subgroup analysis is carried out according to the race, and the random effect model is adopted. No significant differences were found in mixed subgroups (OR 0.839; 95% CI 0.631–1.114) (NIHL cases: 903; control cases: 921) and Asian subgroups (OR 1.026; 95% CI 0.840–1.254) (NIHL cases: 687; control cases: 694), but significant differences were observed in Caucasian subgroups (OR 0.535; 95% CI 0.368–0.779) (NIHL cases: 217; control cases: 227). And no publication bias was observed (*t* = −1.56; *p* = 0.216) (Supplementary Figure S6B). Three studies were included in the rs2763979 (Figure 4F) (NIHL cases: 620; control cases: 915), I^2^ = 64.3%. Using the random effect model, we found no significant correlation (OR 0.982 95% CI 0.712–1.356), and no publication bias was observed (*t* = −0.88; *p* = 0.540) (Supplementary Figure S6C).

#### DNA damage repair related genes

##### Caspase 3

**Figure 5:**
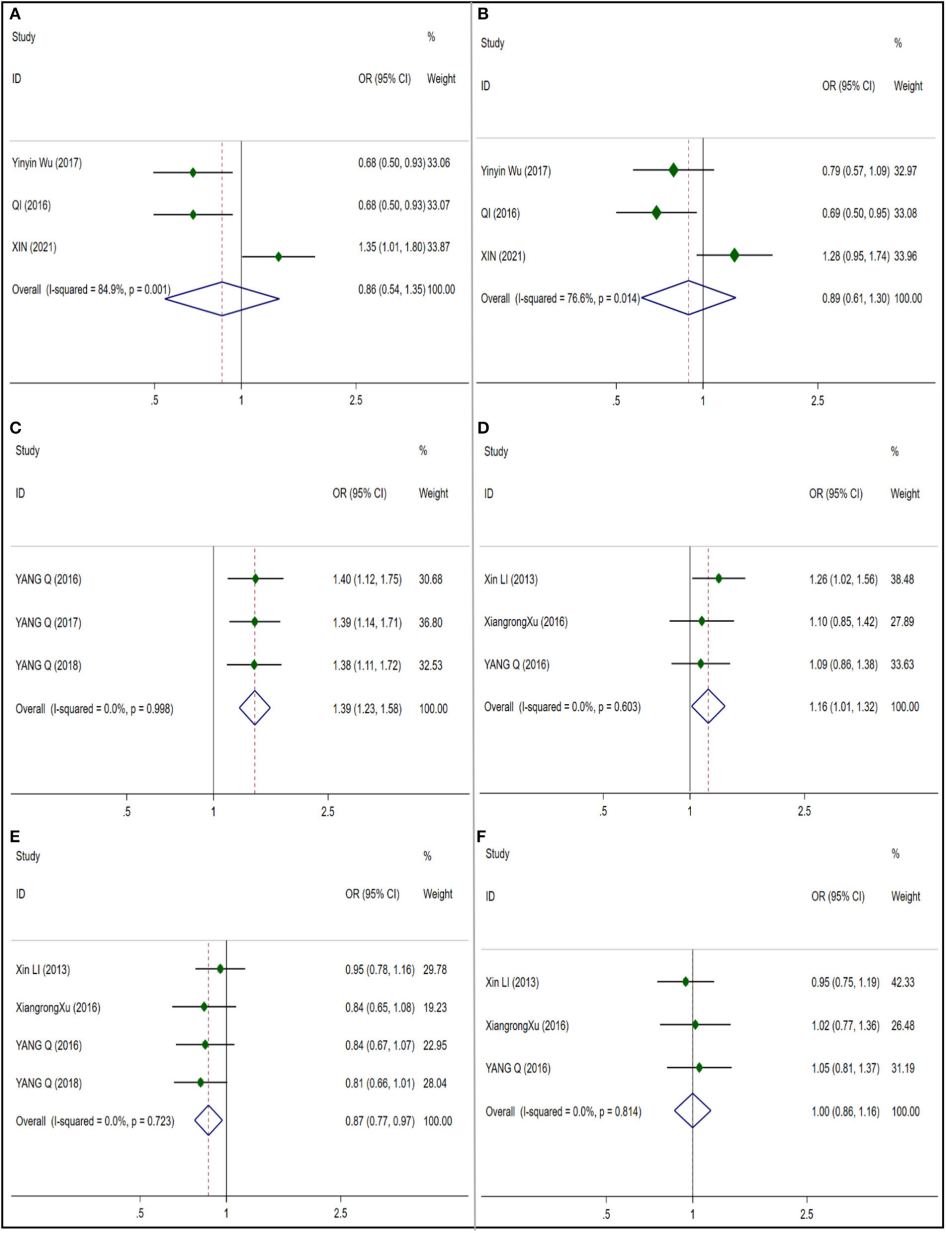
Forest plots for CASP3, EYA4 and GRHL2 polymorphisms. **(A)** CASP3 (rs1049216): allele T; **(B)** CASP3 (rs6948): allele C; **(C)** EYA4 (rs3813346): allele T; **(D)** GRHL2 (rs611419): allele A; **(E)** GRHL2 (rs3735715): allele A; **(F)** GRHL2 (rs3735713): allele A.

We included three studies to analyze the correlation between CASP3 and NIHL, and meta analyzed two gene polymorphisms rs1049216 and rs6948. Among the three articles included by rs1049216 (Figure 5A) (NIHL cases: 851; control cases: 851), using random effect model, no significant correlation was found (OR 0.857; 95% CI 0.542–1.354), and publication bias was observed (*t* = −89.36; *p* = 0.007) (Supplementary Figure S7A). Three articles included in rs6948 (Figure 5B) (NIHL cases: 851; control cases: 851) using random effects model found no significant correlation (OR 0.891; 95% CI 0.611–1.299) and no publication bias was observed (*t* = −3.24; *p* = 0.191) (Supplementary Figure S7B).

##### Eyes absent homolog 4

One gene polymorphism rs3813346 (Figure 5C) (NIHL cases: 961; control cases: 1,253) of EYA4 was analyzed in 3 literatures. Using fixed effect model, a significant correlation (OR 1.392; 95% CI 1.230–1.575) was observed, and no publication bias was observed (*t* = 0.35; *p* = 0.783) (Supplementary Figure S4B).

#### Other noised-induced hearing loss susceptible genes

##### The grainy like 2

**Figure 6:**
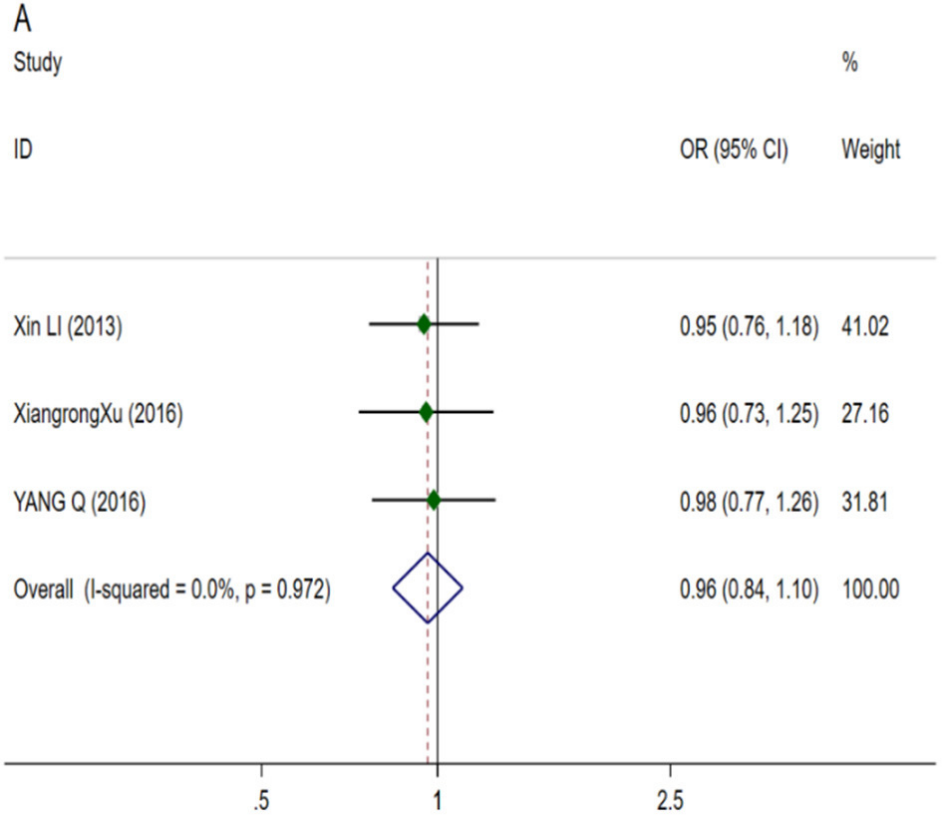
Forest plots for GRHL2. **(A)** GRHL2 (rs3735714): allele T.

We analyzed and evaluated the correlation between rs611419, rs3735715, rs3735713, rs3735714of GRHL2 gene and NIHL susceptibility. In the meta-analysis, I^2^ were all less than 50% and the heterogeneity was low, so using the fixed effect model. We observed a significant correlation (OR 1.157 95% CI 1.012–1.323) in 3 articles included in rs611419 (Figure 5D) (NIHL cases: 865; control cases: 881), which no publication bias was observed (*t* = −1.68; *p* = 0.342) (Supplementary Figure S8A). Four articles were included in the rs3735715 (Figure 5E) (NIHL cases: 1,208; control cases: 1,224), which a significant correlation was observed (OR 0.866; 95% CI 0.774–0.968), and no publication bias was observed (*t* = −1.08; *p* = 0.394) (Supplementary Figure S8B). No significant correlation was observed in the inclusion of 3 articles in rs3735713 (Figure 5F) (NIHL cases: 865; control cases: 881). (OR 0.999; 95% CI 0.863–1.157) and no publication bias (*t* = 1.24; *p* = 0.433) (Supplementary Figure S8C). In the inclusion of 3 articles in rs3735714 (Figure 6A) (NIHL cases: 865; control cases: 881), we did not observe a significant correlation (OR 0.961; 95% CI 0.838–1.104), and no publication bias (*t* = 0.39; *p* = 0.762) (Supplementary Figure S8D).

## Discussion

The purpose of this study was to conduct a systematic review of NIHL-related gene polymorphisms and select a part of the gene polymorphisms which have been extensively studied for meta-analysis. Finally, we performed a meta-analysis of 25 polymorphisms (in 10 genes). Five single nucleotide polymorphisms were found to be significantly associated with the risk of NIHL (one of which was associated only in risk of Caucasian subgroup's NIHL). Our results showed that rs611419 polymorphism and rs3735715 polymorphism (in GRHL2), rs208679 polymorphism (in CAT), rs3813346 polymorphism (in EYA4) was significantly associated with susceptibility to NIHL, and rs2227956 polymorphism (in HSP70) were found to be significantly associated with susceptibility to NIHL in the Caucasian, while the other 20 gene polymorphisms were not found to have significant correlation with NIHL. Thus, we were allowed to identify polymorphisms in screening prediction systems that are more likely to constitute an individual's susceptibility to NIHL in order to be as comprehensive as possible, we thoroughly and extensively searched PubMed, CNKI, Wan Fang, Web of Science, Cochrane library and Embase found 74 papers (involving 64 genes) related to our study. In order to make the analysis results more representative, only polymorphisms with three or more studies were selected for meta-analysis in this study.

NIHL is thought to be possibly caused by pathological changes in the Corti organ, auditory nerve or auditory cortex of the inner ear (Chen et al., 2022), and its pathogenesis involves Antioxidant Genes, Cilia Structure Related Genes, DNA Damage Repair Related Genes, and Other Noised-Induced Hearing Loss Susceptible Genes.

### Antioxidant genes

#### Catalase

From the perspective of metabolic theory, oxidative stress is the key pathological mechanism of NIHL. As a key antioxidant enzyme against oxidative stress, CAT can decompose hydrogen peroxide (H_2_O_2_) and maintain the balance of redox in the body, thus reducing the oxidative damage of cochlea caused by oxidative stress (Chen et al., 2022). Our study found a significant association between catalase polymorphism and NIHL susceptibility: rs208679 polymorphism (in CAT). This study included 1017 NIHL cases and 736 controls. Specifically, the frequency of G allele in rs208679 polymorphism was significantly correlated with NIHL susceptibility. Similarly, such a conclusion can be seen in the study of Wu J. et al. (2022). However, the literature data of rs769217, rs564250 and rs7692143 polymorphisms did not show a significant correlation with NIHL.

#### Glutathione S-transferase

GSTM1, T1 and P1 are also important antioxidant enzymes *in vivo*, which can catalyze the binding of a variety of endogenous or exogenous compounds with reduced glutathione (Chen et al., 2022) and participate in the antioxidant protection of cochlear cells. However, the results of the literature data we included show that there is no significant correlation between GSTM1, GSTT1 and NIHL risk. Taking into account the diversity of ethnic composition of the samples included in the data, we also conducted a subgroup analysis according to ethnicity, which further confirmed that no association between GSTM1 and GSTT1 and NIHL susceptibility was observed in Asian subgroups. The study of Zong et al. (2019) is consistent with our conclusion. Zhou et al. (2014) reported that GSTM1 polymorphism is associated with NIHL in 2014. The reason why the results of this study are inconsistent with our conclusions is that when the heterogeneity *I*^2^ > 50, we used the random effect model, while zhou et al used the fixed effect model. After consulting the information, we still adhere to our effect model selection criteria, and think that more research and larger sample size are needed to get more reliable results.

#### Paraoxonase-2

Paraoxonase-2 has the dual effects of promoting lactase activity and reducing oxidative stress in cells (Altenhöfer et al., 2010). The research data we included do not show that rs7493, rs12026, rs7785846, rs7786401 are related to the risk of NIHL. Cao et al. reported in 2013 that people with the rs7785846 (CT+TT) genotype of the PON2 gene were more likely to have hearing loss when exposed to high noise intensity (Jinglian et al., 2013). In the same year, li et al reported the same conclusion (Xiu-Ting et al., 2013). But Bashmakova et al. (2021) found the opposite in their 2021 study. This contradiction may be caused by ethnic and regional differences in the study samples, which is well worth studying.

#### Superoxide dismutase 2

Superoxide dismutase 2 is an important antioxidant enzyme in the body and the main substance for the body to remove ROS. It may increase the risk of NIHL by affecting the sensitivity of cochlea to noise (Fortunato et al., 2004). However, our results do not support the association between SOD2 and NIHL susceptibility. We have observed that the opposite results have been obtained based on Swedish studies and studies based on Han Chinese (Carlsson et al., 2005; Liu et al., 2010). As found in PON2 data studies, this suggests that genetic polymorphism in SOD2 may lead to race-specific contributions.

#### Cadherin related 23

The tip connections of hair cells convert mechanical sound stimuli into electrical signals. The defect of tip connection leads to hearing loss in humans, which may be proved from a biochemical and genetic point of view (Sakaguchi et al., 2009). The main component of the tip junction is cadherin-associated 23 (CDH23). It plays an important role in cell recognition, migration, tissue differentiation, adult tissue composition and embryonic development (Chen et al., 2022). In this study, we analyzed the polymorphism of rs1227049, rs3802711 and rs1227051 genes in CDH23. From the perspective of included data analysis, there is no significant relationship between these three polymorphisms and NIHL susceptibility. Xin et al observed that the NIHL risk of rs3802711 TT genotype was 2.94 times higher than that of CT+TT genotype in the recessive model, but no correlation between rs3802711 and NIHL risk was found in the allele model (Jiarui et al., 2022). Wu Z-D. et al. (2022) have found the same trend. In our study, we only discussed the correlation under the allele model. In addition, we have added a new case-control study to our analysis of rs1227049 and rs3802711 polymorphisms than previous studies, which makes our results more referential.

#### Heat shock protein genes 70

As a heat shock protein gene, HSP70 can be overexpressed in the inner ear under certain stimulation conditions (including acoustic overstimulation) and play a protective role (Zhang et al., 2020). We observed that the frequency of C allele of rs2227956 polymorphism (in HSP70) was associated with NIHL risk in Caucasian population, but no association was found in mixed population or Han population. This may indicate that genetic polymorphism is different in Different races.

### DNA damage repair related genes

#### Caspase 3

The activation of caspase family is the central link leading to apoptosis, in which CASP3 and CASP7 are the main executors. Caspase3 gene polymorphism is closely related to tumor (Yan et al., 2013). The correlation between them and NIHL is not clear, and there is no significant correlation between them in the data analysis we included.

#### Eyes absent homolog 4

Eye deletion homolog 4 not only plays a role in regulating tissue-specific differentiation during embryonic development, but also participates in a variety of biological activities, including maintaining the development and maturation of Corti organs (Chen et al., 2022). This study included 961 patients with NIHL and 1,253 controls to analyze the relationship between rs3813346 polymorphism and NIHL susceptibility. We observed a significant correlation. Although the inclusion process meets our pre-set inclusion criteria and there is no publication bias, the three papers included come from the same author and the sample sources are very similar, so we have reservations about this association. We need more samples and more central research to verify this result.

### Other noised-induced hearing loss susceptible genes

#### The grainy like 2

GRHL2, also known as the homologous gene of mammalian granular head (BOM) and T transcription factor cell promoter 2-Like3 (TFCP2L3) (Zhang et al., 2020), is a transcription factor related to the composition of Corti organs (Li et al., 2020). It plays a key role in embryonic development and maintenance of epithelial cells (Chen et al., 2022). This study found that rs611419 polymorphism and rs3735715 polymorphism (in GRHL2) were significantly associated with NIHL susceptibility. The rs611419 polymorphism analysis includes 865 NIHL case samples and 881 control samples. To our knowledge, our study is the first meta-analysis to evaluate and demonstrate a potential association between rs611419 polymorphism and NIHL susceptibility. 1,208 NIHL cases and 1,224 controls were included in the analysis of rs3735715 polymorphism, and the correlation between rs3735715 and NIHL risk was observed. Li et al. (2020) found that the rs3735715 A T/TT genotype was more protective against NIHL than the AA genotype. This is consistent with our findings.

Through a large number of literature search and systematic review, 10 genes and 25 single nucleotide polymorphisms were selected for meta-analysis. The quality of the literature we included is generally high, which provides a more reliable basis for our research results. To some extent, this meta-analysis deals with the contradiction between different research results and tries to understand the role of these genes in NIHL susceptibility from the perspective of the pathogenesis of NIHL, but there are still many gene polymorphisms have not been included in the study. There are three main reasons. First of all, the genetic polymorphism related to NIHL is only the tip of the iceberg, and there is still a lot of room for discovery and research of related genes. Second, many gene polymorphism studies are insufficient and are generally conducted in a single-center study. And in order to ensure that our research results are more valuable and efficient, only gene polymorphisms that have been reported by at least 3 literatures can be studied by us. Third, only English literature, Chinese literature and one Russian literature are included in our study, and other languages are not taken into account. In addition, the current research on NIHL susceptibility genes, especially experimental research, has some difficulties. The pathogenesis of NIHL is a problem induced by multiple factors, which is often affected by ethnic differences, regional differences, sample population differences and other factors. What's more, the study of genes always involves family, heredity and other issues, but it is obviously not in line with medical ethics if the subjects are exposed to noise. So we still face great challenges in practical research.

## Conclusion

As we discussed, genetic factors are involved in the pathogenesis of NIHL in a variety of ways, affecting the damage and protection of cochlear hair cells and Corti organs, and there is no treatment for hair cell and Corti organ damage (Kim et al., 2018). Therefore, the prevention of NIHL is very important. Screening genes related to NIHL susceptibility and providing an effective risk prediction system for people, especially high-risk groups, can better identify and prevent the occurrence of NIHL. We believe that with the deepening of people's understanding of NIHL and the continuous updating and development of new gene research methods, more and more genes related to NIHL will be found.

## Data availability statement

The original contributions presented in the study are included in the article/Supplementary material, further inquiries can be directed to the corresponding author.

## Author contributions

LW, HW, YX, and FXia were suitable for the study design, literature searches, statistical analysis, and manuscript preparation. QZ and FXio were supervised this study. All authors contributed to the article and approved the submitted version.
