# Supplementary material for: Polymorphisms and NIHL: a systematic review and meta-analyses
Source: Front Cell Neurosci. 2023 Jun 15;17:1175427. doi: 10.3389/fncel.2023.1175427 (PMC10309027; doi:10.3389/fncel.2023.1175427)
Supplement: Supplementary file 1 [file Data_Sheet_1.docx]

***Supplementary Material***

**Polymorphisms and NIHL: A Systematic Review and Meta-Analyses**

**Lu Wang^1^, HanYu Wang^1^, Feng Xiang^1^, YuLu Xiang^3^, Feng Xiong^1^, QinXiu Zhang^1, 2*^**

^1^Clinical Medical College, Chengdu University of Traditional Chinese Medicine, Chengdu, Sichuan, China

^2^School of Medical and Life Sciences, Chengdu University of Traditional Chinese Medicine, Chengdu, Sichuan, China

^3^The Affiliated Hospital of Inner Mongolia Medical University, Inner Mongolia, China

*** Correspondence:**

QinXiu Zhang^1, 2*^

[zhqinxiu@163.com](mailto:zhqinxiu@163.com)

**Supplementary Figures**


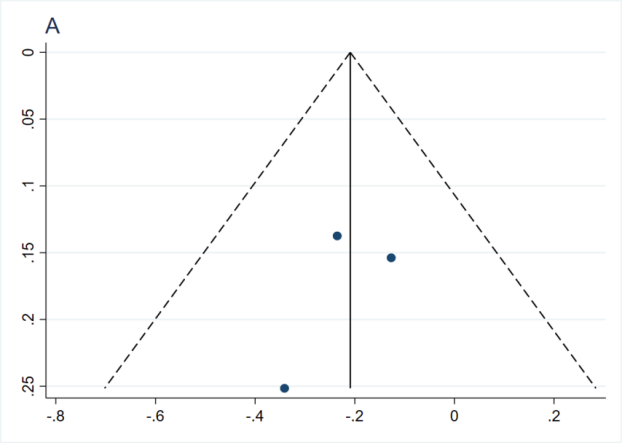

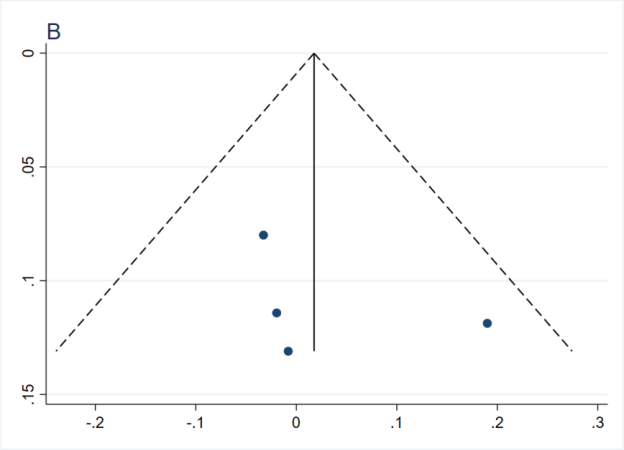

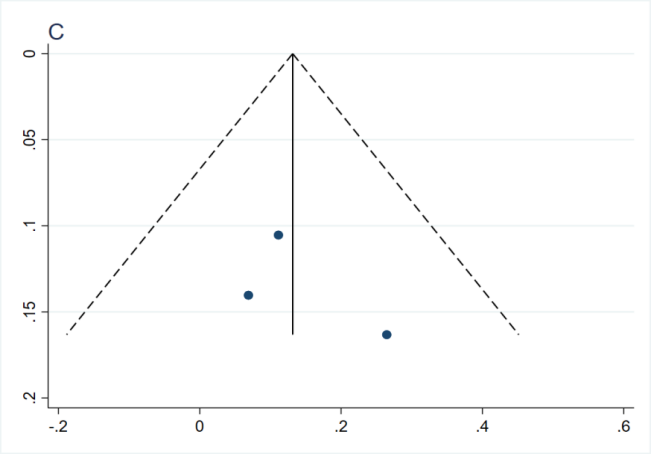

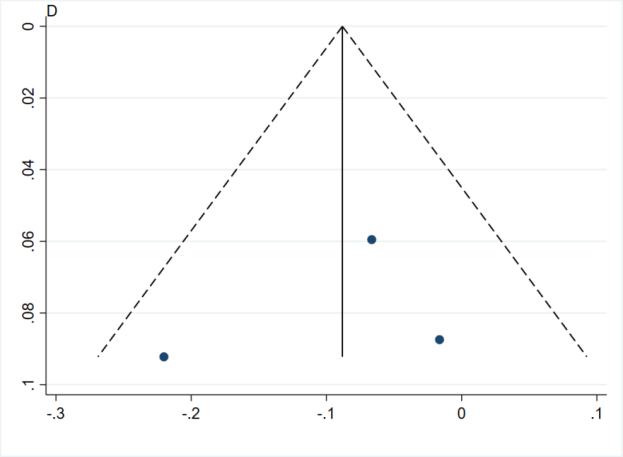


**Supplementary Figure 1.** Funnel plot of (A) CAT (rs208679); (B) CAT (rs769217); (C) CAT (rs564250); (D) CAT (rs769214).


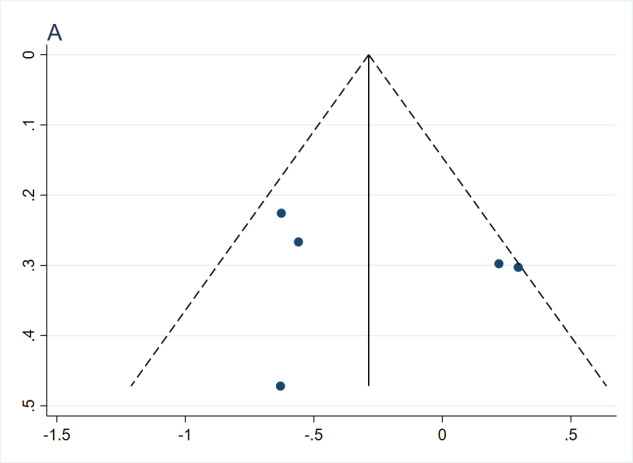

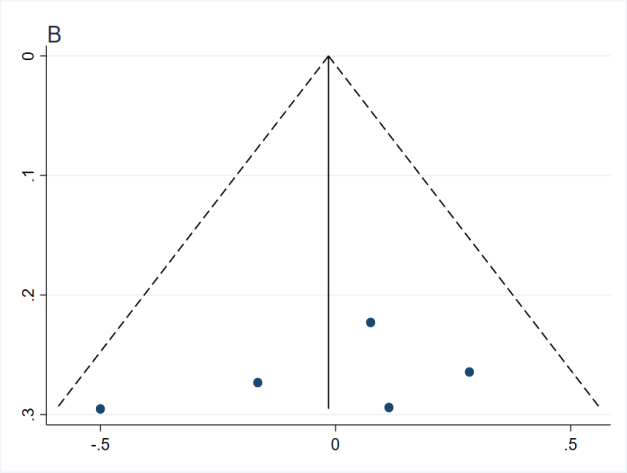


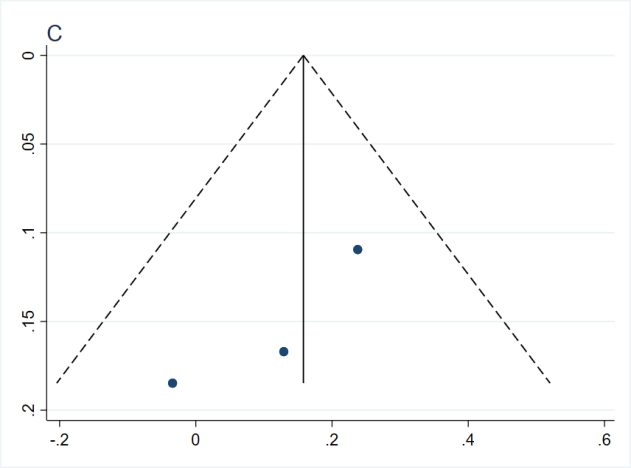


**Supplementary Figure 2.** Funnel plot of (A) GST (M1); (B) GST (T1); (C) CAT (rs1695).


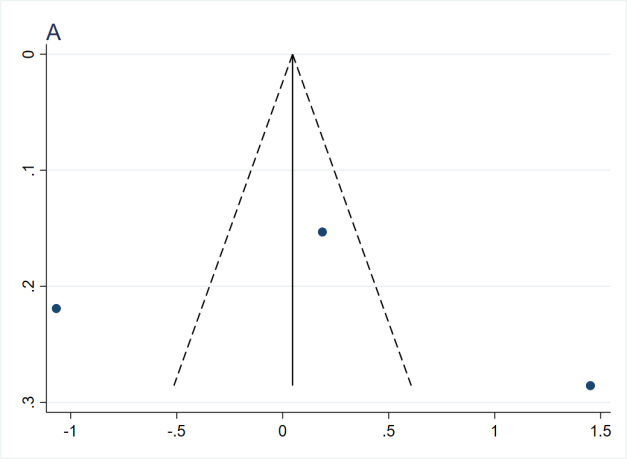

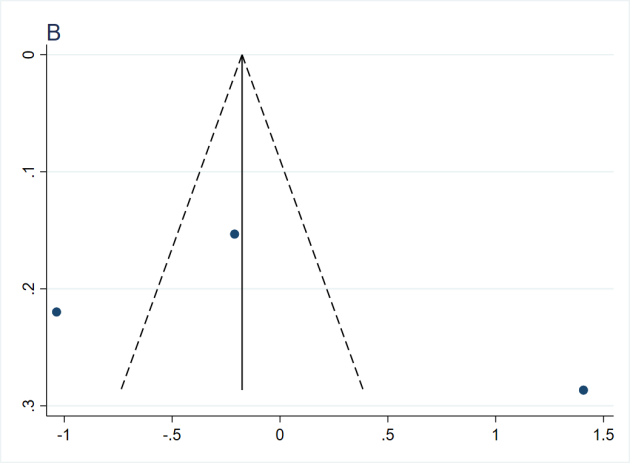

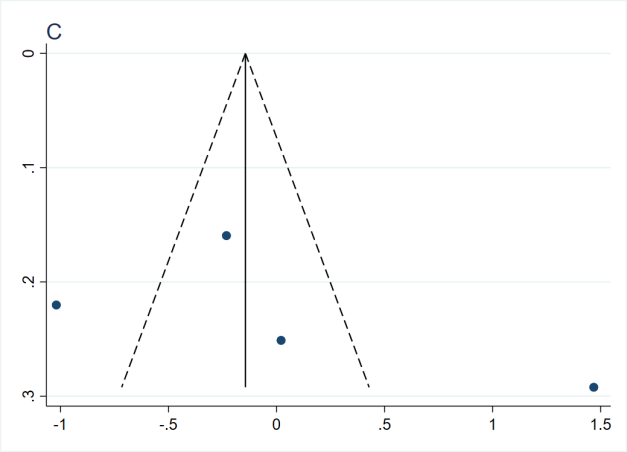

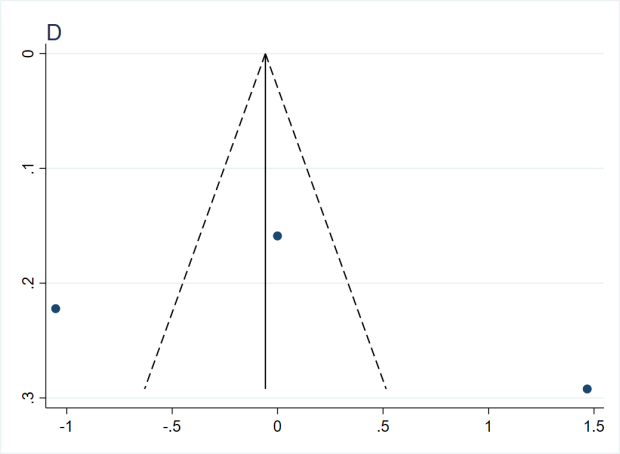


**Supplementary Figure 3.** Funnel plot of (A) PON2 (rs7493); (B) PON2 (rs12026); (C) PON2 (rs7785846); (D) PON2 (rs7786401).


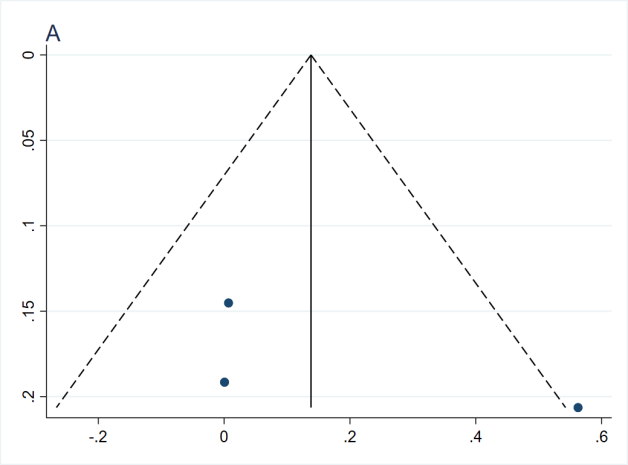

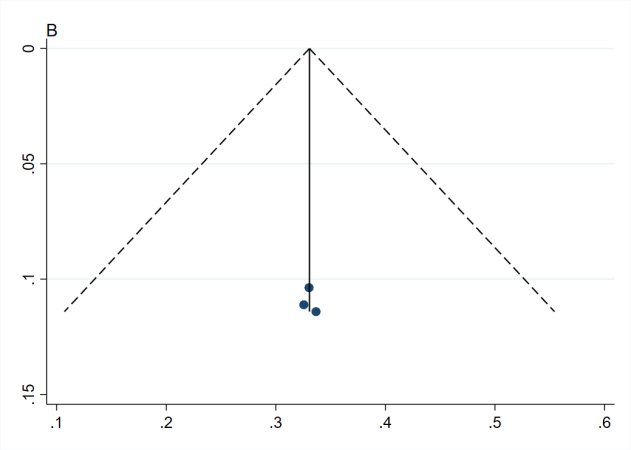


**Supplementary Figure 4.** Funnel plot of (A) SOD2 (rs4880); (B) EYA4 (rs3813346).


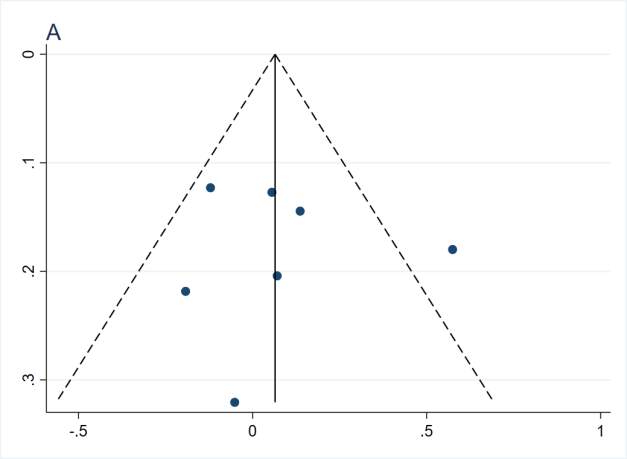

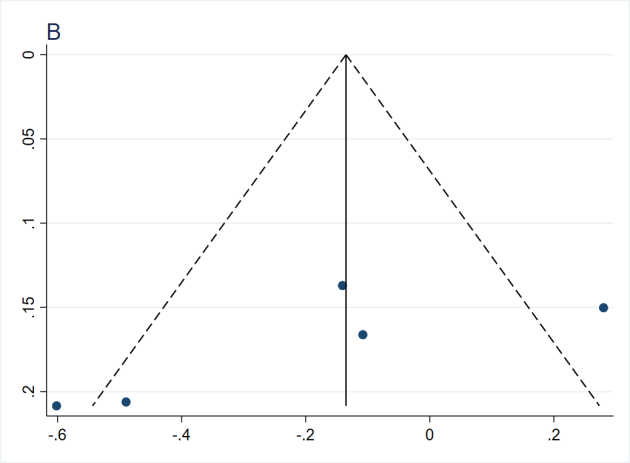

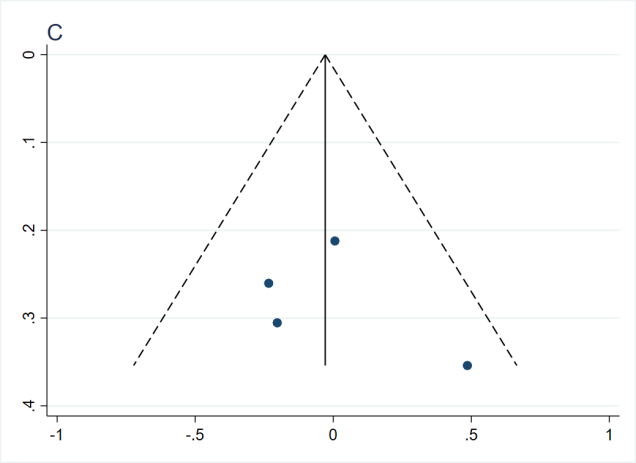


**Supplementary Figure 5.** Funnel plot of (A) CDH23 (rs1227049); (B) CDH23 (rs3802711); (C) CDH23 (rs1227051).


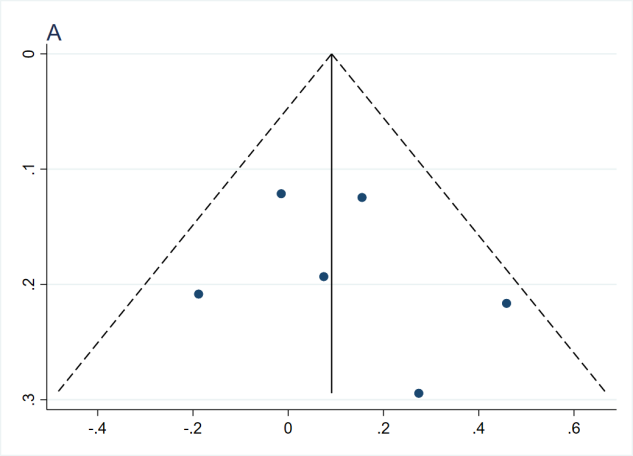

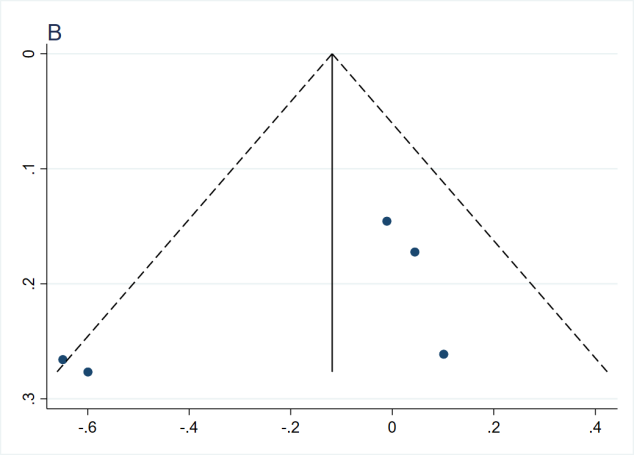

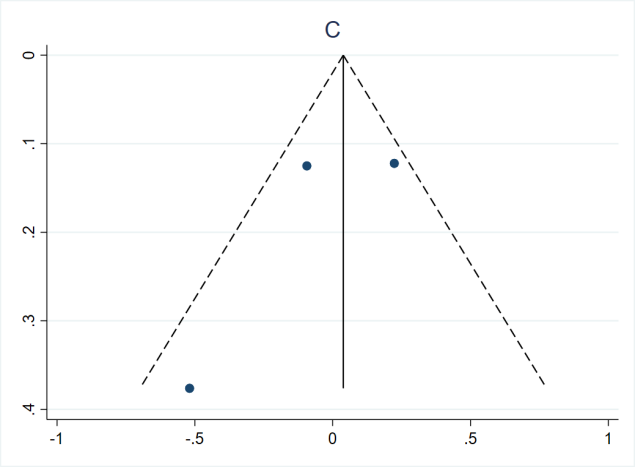


**Supplementary Figure 6.** Funnel plot of (A) HSP70 (rs1043618); (B) HSP70 (rs2227956); (C) HSP70 (rs2763979).


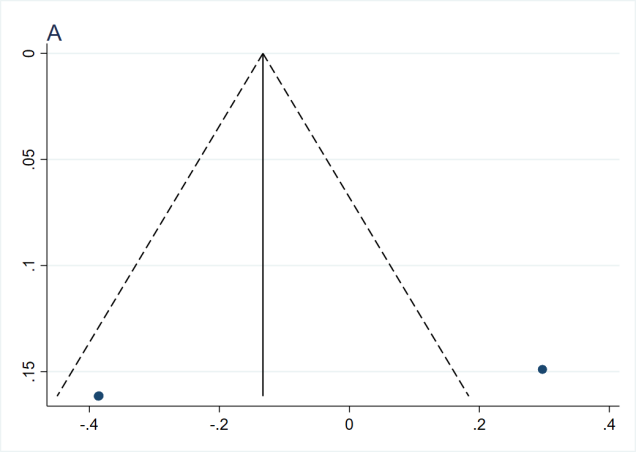

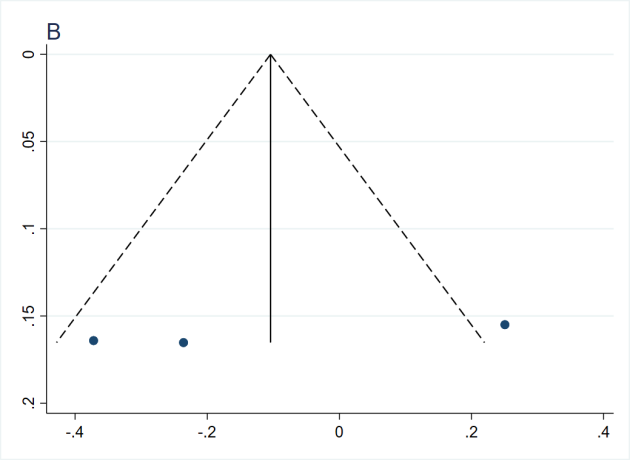


**Supplementary Figure 7.** Funnel plot of (A) CASP3 (rs1049216); (B) CASP3 (rs6948).


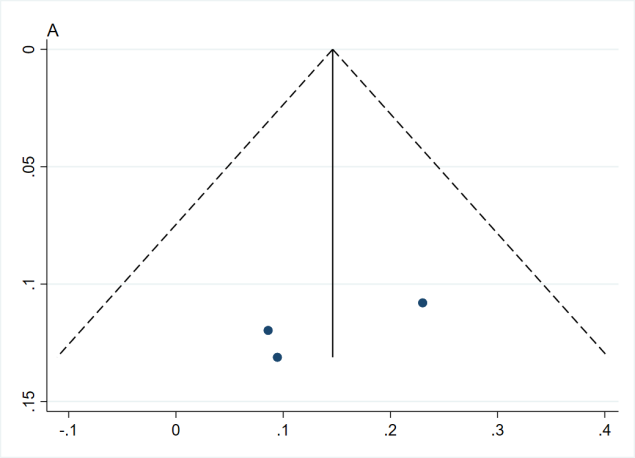

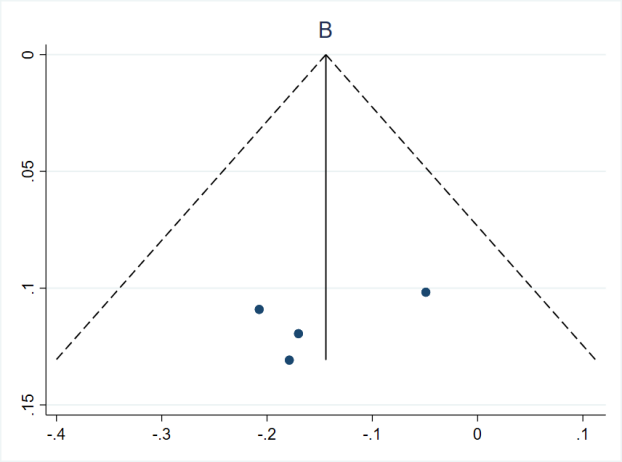

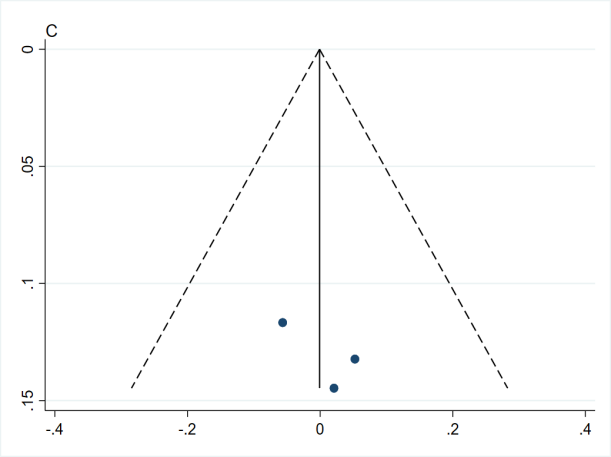

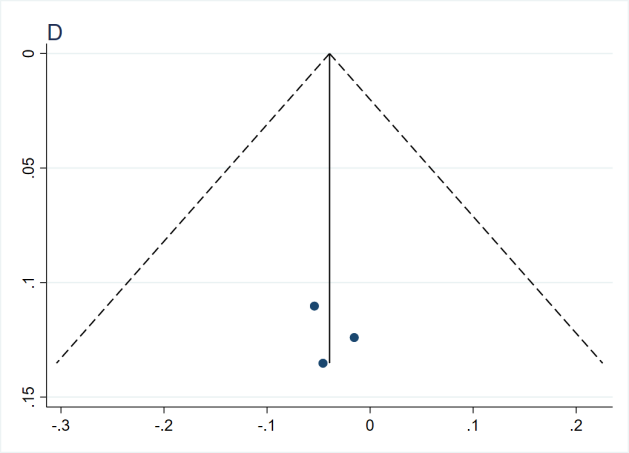


**Supplementary Figure 8.** Funnel plot of (A) GRHL2 (rs611419); (B) GRHL2 (rs3735715); (C) GRHL2 (rs3735713); (D) GRHL2 (rs3735714).
