# Supplementary material for: Polymorphisms and NIHL: a systematic review and meta-analyses
Source: Front Cell Neurosci. 2023 Jun 15;17:1175427. doi: 10.3389/fncel.2023.1175427 (PMC10309027; doi:10.3389/fncel.2023.1175427)
Supplement: Supplementary file 2 [file Table_1.docx]

Supplementary Material

**Polymorphisms and NIHL: A Systematic Review and Meta-Analyses**

**Lu Wang^1^, HanYu Wang^1^, Feng Xiang^1^, YuLu Xiang^3^, Feng Xiong^1^, QinXiu Zhang^1, 2*^**

^1^Clinical Medical College, Chengdu University of Traditional Chinese Medicine, Chengdu, Sichuan, China

^2^School of Medical and Life Sciences, Chengdu University of Traditional Chinese Medicine, Chengdu, Sichuan, China

^3^The Affiliated Hospital of Inner Mongolia Medical University, Inner Mongolia, China

*** Correspondence:**

QinXiu Zhang^1, 2*^

[zhqinxiu@163.com](mailto:zhqinxiu@163.com)

**Supplementary Table 1** **Retrieval strategy sample of PubMed**

| #1 | Polymorphism, Single Nucleotide [MeSH terms] |
| --- | --- |
| #2 | Nucleotide Polymorphism, Single [text word] |
| #3 | Nucleotide Polymorphisms, Single [text word] |
| #4 | Polymorphisms, Single Nucleotide [text word] |
| #5 | Single Nucleotide Polymorphisms [text word] |
| #6 | SNPs [text word] |
| #7 | Single Nucleotide Polymorphism [text word] |
| #8 | Or/#1-7 |
| #9 | Hearing Loss, Noise-Induced [MeSH terms] |
| #10 | Hearing Loss, Noise Induced [text word] |
| #11 | Noise-Induced Hearing Loss [text word] |
| #12 | Acoustic Trauma [text word] |
| #13 | Or/#9-12 |
| #14 | Case Reports [Publication Type] |
| #15 | Comment [Publication Type] |
| #16 | Congress [Publication Type] |
| #17 | Consensus Development Conference [Publication Type] |
| #18 | Duplicate Publication [Publication Type] |
| #19 | Editorial [Publication Type] |
| #20 | English Abstract [Publication Type] |
| #21 | Observational Study, Veterinary [Publication Type] |
| #22 | Retracted Publication [Publication Type] |
| #23 | Retraction of Publication [Publication Type] |
| #24 | Meta-Analysis [Publication Type] |
| #25 | Review [Publication Type] |
| #26 | Systematic Review [Publication Type] |
| #27 | Or/#14-24 |
| #28 | Humans [Species] |
| #29 | #8 and #13 not #27 and #28 |
| ((("polymorphism, single nucleotide"[MeSH Terms] OR "nucleotide polymorphism single"[Text Word] OR "nucleotide polymorphisms single"[Text Word] OR "polymorphisms single nucleotide"[Text Word] OR "single nucleotide polymorphisms"[Text Word] OR "SNPs"[Text Word] OR "single nucleotide polymorphism"[Text Word]) AND ("hearing loss, noise induced"[MeSH Terms] OR "hearing loss noise induced"[Text Word] OR "noise induced hearing loss"[Text Word] OR "acoustic trauma"[Text Word])) NOT ("case reports"[Publication Type] OR "Comment"[Publication Type] OR "Congress"[Publication Type] OR "consensus development conference"[Publication Type] OR "duplicate publication"[Publication Type] OR "Editorial"[Publication Type] OR "english abstract"[Publication Type] OR "observational study, veterinary"[Publication Type] OR "retracted publication"[Publication Type] OR "retraction of publication"[Publication Type] OR "Meta-Analysis"[Publication Type] OR "Review"[Publication Type] OR "systematic review"[Publication Type])) AND (humans[Filter]) | |
